# Including family and friend care partners in the arts in long-term care

**DOI:** 10.1093/geroni/igag081

**Published:** 2026-07-10

**Authors:** Eden R Champagne, Erica MacTavish, Debra Chandler, Lia Tsotsos, Kate Dupuis

**Affiliations:** Sheridan Centre for Elder Research, Sheridan College, Oakville, Ontario, Canada; Sheridan Centre for Elder Research, Sheridan College, Oakville, Ontario, Canada; Department of Recreation and Leisure Studies, University of Waterloo, Waterloo, Ontario, Canada; Concerts in Care Ontario, Toronto, Ontario, Canada; Sheridan Centre for Elder Research, Sheridan College, Oakville, Ontario, Canada; Generator, Sheridan College, Oakville, Ontario, Canada; Sheridan Centre for Elder Research, Sheridan College, Oakville, Ontario, Canada; Schlegel-UW Research Institute for Aging, Waterloo, Ontario, Canada

**Keywords:** Long-term care, Creativity, Caregivers

The number of people living with dementia worldwide is increasing. The majority of people living with dementia are supported to live at home by family and friend care partners (FFCPs). Importantly, FFCPs often remain highly involved in care decisions even once the people living with dementia move into long-term care (LTC), and they can continue to experience burden, identity strain, and grief even posttransition to LTC (Ontario Caregiver Association, 2025). Accordingly, the LTC setting presents a unique opportunity to support both people living with dementia and their FFCPs. Engaging in the creative arts can enhance all domains of health and well-being (e.g., Fancourt & Finn, 2019), yet published studies on arts-based opportunities for dyadic connection between people living with dementia and their FFCP remain limited (Bourne et al., 2021; Durocher et al., 2022), suggesting they are not yet broadly embedded in LTC practice. Rather, FFCPs are encouraged to join as a “plus-1” or observer, rather than to purposely participate for their own benefit. In this *Innovation Note*, we present a novel approach in which FFCPs can meaningfully benefit from purposeful participation in creative activities in LTC. Our proposed innovation is intentional changes to the physical spaces of LTC to support creative connections, modifying the culture of care, purposefully creating arts-based programs which include FFCPs, and studying the benefits of this intersection (see Figure 1).

**Figure 1:**
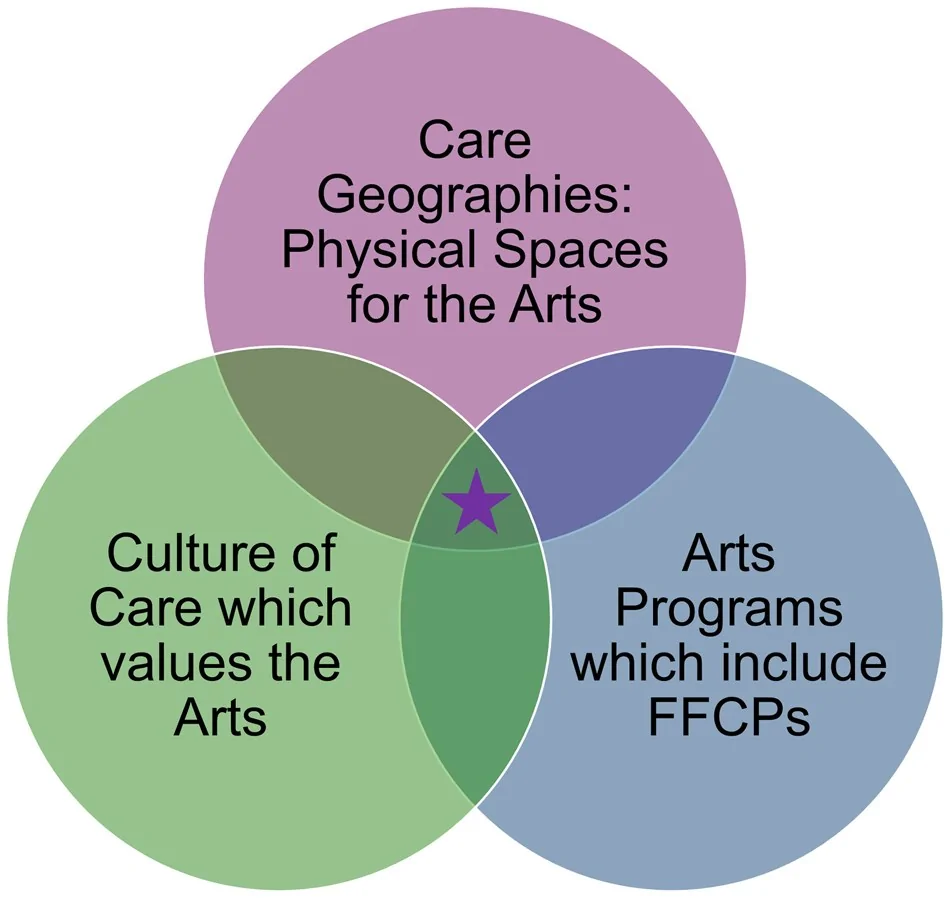
Fruitful areas for innovation: including family and friend care partners (FFCPs) in the arts in long-term care.

## Domain 1: care geographies—if you build it, they can create

Despite a substantial literature demonstrating the benefits of creative engagement for people living with dementia and their FFCPs, care homes have been slow to integrate these approaches into their physical environments. Few LTC homes have purpose-built spaces designed to support and encourage arts participation. Often, arts programs are shoehorned into existing spaces, such as moving lunch tables aside for a concert or painting at one end of a table in a busy multipurpose space. Practical implementation considerations include developing, retrofitting, or claiming a space within the LTC home that can be intentionally used for arts programming. Staff can be educated on the importance of having art materials (e.g., paint, musical instruments) available for residents to engage with in a more “free-range” manner. These changes may lead to benefits for formal care staff and FFCPs, such as reduced burden, enhanced communication, and feelings of pride and connection. Creating such spaces in LTC will require shifts in policies and priorities from local governments and LTC leadership.

## Domain 2: culture of care—embracing the arts

While there has been a prominent cultural shift toward “person-centered care” in LTC, arts activities that are tailored to the unique interests of residents and FFCPs are underdeveloped. For example, research has explored the benefits of group concerts in LTC (Dupuis & Chandler, 2024), but individuals often enjoy different genres of music. Personalized music interventions, such as creating unique playlists for dyads to listen to together (Bufalini et al., 2022), could be beneficial, but the wide-scale adoption of these approaches is unknown. In addition, in a LTC culture that truly values the arts, care staff can support creative communication, such as by making sure that materials are available when FFCPs visit to support interactions. Yet, recent findings suggest that health care workers may lack knowledge or have incorrect knowledge about implementing arts programming (Van Lith et al., 2026), indicating a need for staff education initiatives.

## Domain 3: program changes to include FFCPs

FFCPs often neglect their own leisure and well-being due to care responsibilities and care strain (e.g., Fortune & Gauthier-Gilbert, 2025). Providing FFCPs with creative arts programming when they visit LTC represents an innovative strategy to support their well-being. A recent scoping review by our team (Champagne et al., manuscript under review) found that, across three international databases, only seven published studies had explored arts programs in LTC that intentionally included FFCPs for their own benefit. Our review suggests that when arts programs include FFCPs, they can provide meaningful moments of connection and can help FFCPs look forward to LTC visits (Champagne et al., manuscript under review). Music listening (e.g., a 15-min session in the resident’s room) and collaborative songwriting are examples of programs that could have dyadic and personal benefits. Importantly, opportunities to participate in arts activities may vary across cultures, socioeconomic contexts, and FFCP availability. Efforts to accommodate these diverse experiences are crucial.

## Conclusion and call to action

In sum, given the known challenges that FFCPs often face, it is crucial to develop, implement, and evaluate innovative strategies to support them. The call to action for LTC leaders and staff, policy makers, and researchers is to create intentional spaces for the arts, embrace person-centered creativity, and prioritize and evaluate arts-based programs that include FFCPs. Ideally, personal and dyadic well-being could be enhanced when these processes intersect and are enacted in tandem.

## Data Availability

Given the nature of this Innovation Note, no data were collected for this publication.
